# Adolescent talus body fracture with high displacement: A case report

**DOI:** 10.1097/MD.0000000000012043

**Published:** 2018-08-21

**Authors:** Shunpei Hama, Ryu Onishi, Masataka Yasuda, Kenta Minato, Masahiro Miyashita

**Affiliations:** Department of Orthopaedic Surgery, Baba Memorial Hospital, Osaka, Japan.

**Keywords:** adolescent, arthrosis, avascular necrosis, talus fracture

## Abstract

**Rationale:**

Talus fracture is relatively rare in adults. Furthermore, talus fracture in pediatric population is rarer than in adult population. Although undisplaced talus fractures can be treated conservatively, most of talus fractures with displacement require surgical treatment in both pediatric and adult patients. In addition, avascular necrosis and arthrosis are the main complications of displaced talus fracture.

**Patient concerns:**

A 14-year-old boy was referred to our hospital owing to foot injury sustained on jumping off about 10 stairs.

**Diagnosis:**

Highly displaced talus body fracture of the dome and the posterior process.

**Interventions:**

Because the Linhart classification of this case was III-C and instability at the fracture site persisted even after closed reduction, we performed arthroscopic-assisted reduction and internal fixation (ARIF) using headless screws and an external fixator under general anesthesia.

**Outcomes:**

We removed the external fixator at 3 months after the surgery. At the 1-year follow-up, the patient was able to walk with full weight bearing and his Japanese Orthopaedic Association score recovered from 9 points before the surgery to 95 points. The range of motion of dorsiflexion and plantarflexion was 10° and 60°, respectively, which were similar to that on the left side. No signs of bone necrosis or arthrosis were observed on imaging.

**Lessons:**

ARIF with external fixation might be the treatment of choice for such a case.

## 1 Introduction

Talus fractures are relatively rare and account for approximately 1% of all fractures.^[1]^ Talus body fractures account for 13% to 23% of all talus fractures and are less common compared with the talus neck fractures.^[2]^ In addition, talus body fractures are at a higher risk of avascular necrosis (AVN).^[3]^ Although talus body fractures can be treated conservatively by using short leg casting if they are undisplaced, most of displaced fractures need surgical treatment.^[4]^

Literature pertaining to pediatric talus fracture is relatively scarce. The incidence of talus fracture in the pediatric population varies between 0.01% and 0.08% compared with 0.3% in adults.^[5,6]^ We report the case of an adolescent patient who had a highly displaced talus body fracture. The patient and his parents consented to this case report being submitted for publication. The study was approved by Baba Memorial Hospital Review Board.

## 2 Case report

**Figure 1:**
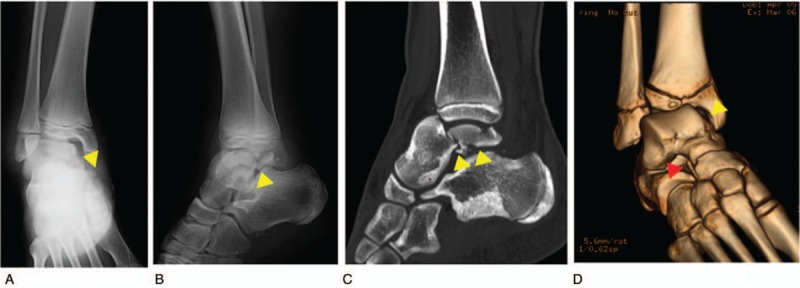
Preoperative radiographs and computed tomography (CT) (yellow arrow head: talus body fracture; red arrow head: avulsion fracture of navicular bone). (A) Anterior-posterior view. (B) Lateral view. (C) Sagittal image of CT. (D) 3-Dimensional reconstruction of CT.

**Figure 2:**
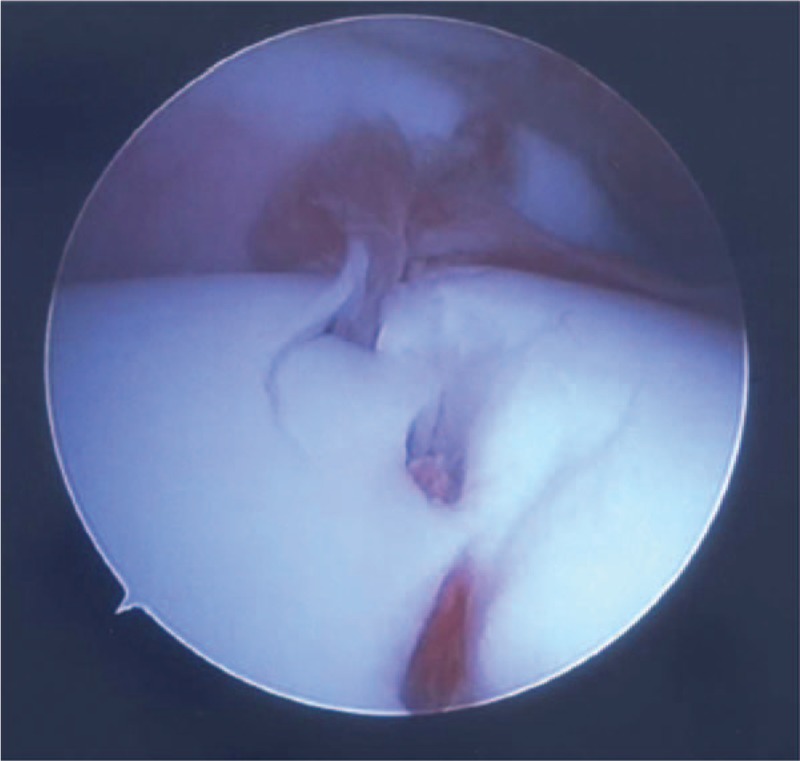
Arthroscopic findings.

**Figure 3:**
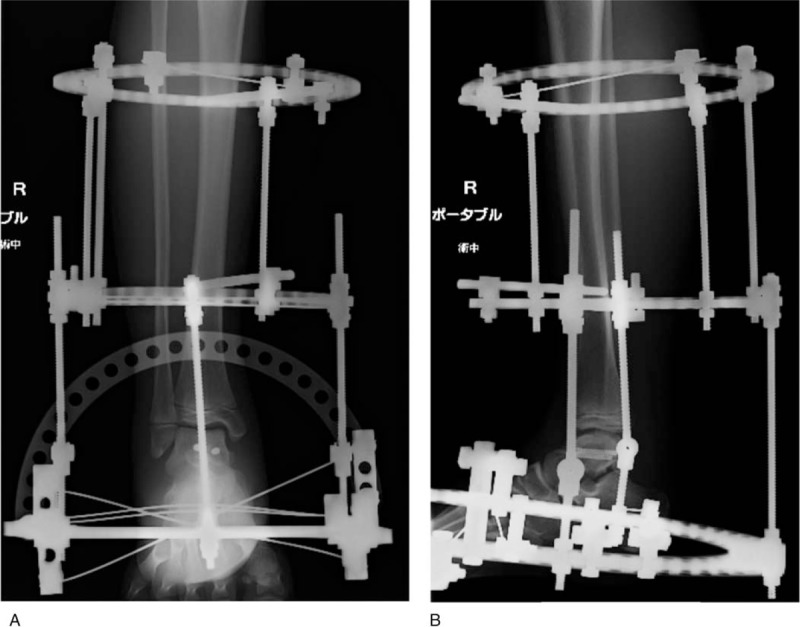
Postoperative radiographs. (A) Anterior-posterior view. (B) Lateral view.

**Figure 4:**
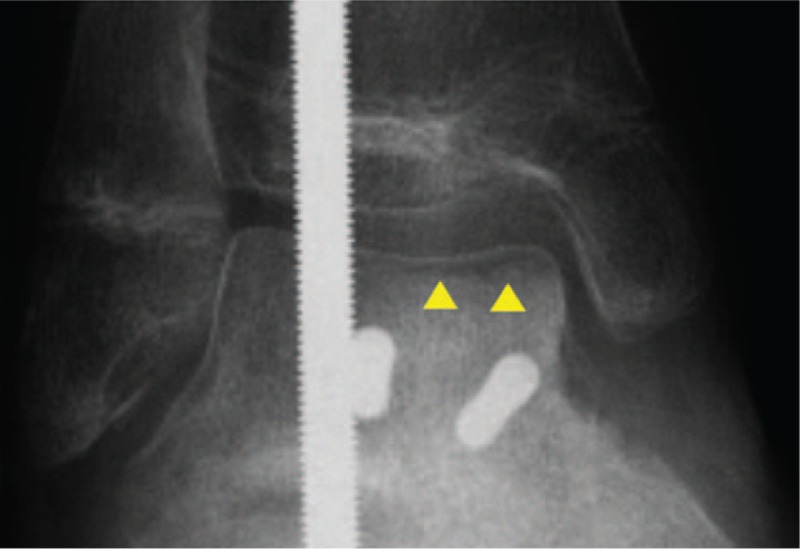
Anterior-posterior radiograph showing subchondral radiolucent band in the talus dome (arrow head).

**Figure 5:**
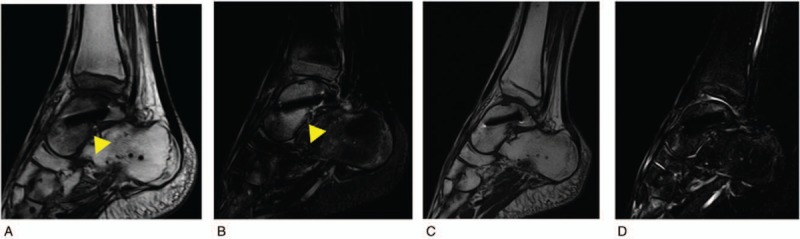
Sagittal images of magnetic resonance imaging (MRI) at 3 and 6 months after the surgery. (A) T1-weighted image at 3 months after the surgery. (B) T2-weighted short-tau invasion recovery (STIR) image at 3 months after the surgery. Dome of talus shows low intensity on T1-weighted image and high intensity on T2-weighted STIR image (arrow head). (C) T1-weighted image at 6 months after the surgery. (D) T2-weighted STIR image at 6 months after the surgery. Low intensity of the dome has changed to iso-intensity on T1-weighted image and high intensity on T2-weighted STIR image has also changed to iso-intensity.

**Figure 6:**
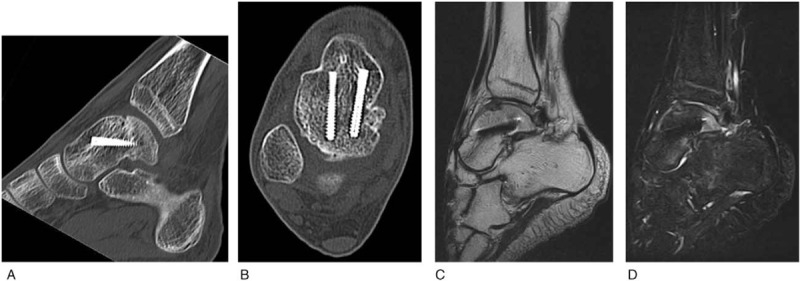
Computed tomography (CT) and magnetic resonance imaging (MRI) at 1 year after the surgery. (A) Sagittal image of CT. (B) Axial image of CT. (C) T1-weighted image of MRI. (D) T2-weighted short-tau invasion recovery image of MRI. The talus fracture has healed and no signs of avascular necrosis or arthrosis are seen.

A 14-year-old boy was referred to our hospital owing to foot injury sustained on jumping off about 10 stairs. Physical examination showed swelling and tenderness around his right ankle. Radiography and computed tomography (CT) showed a highly displaced talus body fracture of the dome and the posterior process and avulsion fracture of navicular bone (Fig. 1). Closed reduction was performed under the sciatic nerve and saphenous nerve block on the same day and the plaster cast was used to prevent displacement. Because the Linhart classification^[6]^ of this case was III-C and instability persisted, we planned the surgery. Four days after the injury, we performed arthroscopic-assisted reduction and internal fixation (ARIF) using headless screws (Acutrak Standard; Acumed, Hillsboro, OR) and an external fixator (TrueLoK Ring Fixation System; Orthofix GmbH, Ottobrunn, Germany) under general anesthesia. First we maintained the reduced position by setting the external fixator. Then we inserted 2 headless screws in an anteroposterior direction percutaneously by compressing the back of the talus with Kirshner wires curved like olive wires. Before the insertion of the screws, we confirmed that the step off was almost reduced by arthroscopy (Fig. 2). Figure 3 shows the postoperative radiographs. At the 8-week follow-up, we observed Hawkins sign (Fig. 4) in the anteroposterior radiograph. We confirmed bone union by CT at the 3-month follow-up and removed the external fixator. After the removal of the external fixator, we advised the patient to walk with a patella tendon-bearing ankle foot orthosis. Magnetic resonance imaging (MRI) after the removal of the external fixator revealed hyperemia of the talus body (Fig. 5A,B). MRI at the 6-month follow-up showed resolution of hyperemia and the bone signal was restored to normal intensity (Fig. 5C, D). Partial weight-bearing walking was then initiated. He was able to walk with full weight bearing 8 months after the operation. At the 1-year follow-up, his Japanese Orthopaedic Association score recovered from 9 points before the surgery to 95 points. The range of motion of dorsiflexion and plantarflexion was 10° and 60°, respectively, which were similar to that on the left side. No signs of bone necrosis or arthrosis were observed on imaging (Fig. 6).

## 3 Discussion

Talus fracture is very rare in children. The typical mechanism of fracture is dorsiflexion and axial loading; therefore, talus neck is the most common site of fracture.^[7]^ Our patient sustained fractures of the dome and posterior process of talus body. Therefore, the patient might sustain the fracture in the plantarflexion position and by axial loading. Although talus fractures in children are less vulnerable to displacement owing to thick periosteum and abundant malleable cartilage, our patient had a highly displaced fracture.^[7]^ Eberl et al compared talus fractures of children younger than 12 years and those of adolescents older than 12 years. They found that adolescents present with more severe fractures compared with children.^[8]^ Therefore, the patient's age and high-energy mechanism may have contributed to the high displacement of talus fracture in this case.

Undisplaced talus body fracture can be treated by nonoperative treatment including immobilization with short leg casting. However, talus body fracture with displacement often needs surgical treatment. The main goal of treatment is restoration of articular surface and alignment.^[4]^ This strategy is applicable to both adult and pediatric patients.^[8]^ In this case, the talus body fracture so highly displaced that we had to perform closed reduction. Furthermore, instability remained after the reduction. Therefore, we planned the surgery. ARIF was thought to be desirable for the intra-articular fracture like the present case.

The fragments of the dome and the posterior process were very small. We used screws in addition to the external fixator because the sole use of screws may not have completely stabilized the fracture. We inserted the headless screws anteroposteriorly and achieved good fracture healing; however, we probably should have inserted the screws in a posteroanterior direction, because it is easier to insert a screw from a small fragment than to the small fragment.

The most important complications of talus fractures include AVN and arthrosis. AVN and arthrosis are more frequently associated with a high-energy injury and displaced fractures.^[7]^

Talus has little muscular or tendinous attachments and there is little redundancy in the 3 primary arteries which supply the talus, that is, artery of the tarsal canal, artery of the tarsal sinus, and deltoid artery.^[7]^ Therefore, the talus is particularly vulnerable to ischemia and AVN if its vascular supply is disturbed by injury including fractures. Smith et al reviewed the literature pertaining to pediatric talus fractures; they reported that the incidence of posttraumatic AVN ranges from 0% to 66% in children.^[7]^ Hawkins sign^[9]^ is useful to rule out AVN and refers to the subchondral radiolucent band in the talar dome in the anteroposterior view of radiograph at 6 to 8 weeks after injury. Subchondral low bone density is believed to reflect reactive hyperemia and vitality of the talus. The reported sensitivity and specificity of Hawkins sign is 100% and 57.7%, respectively.^[10]^ According to Pearce et al, MRI is the most sensitive technique for detection of AVN in the early stages and in cases in which there is a high clinical suspicion of AVN based on X-ray radiographs.^[11]^ Talus body fractures have a higher prevalence of AVN.^[3]^ This is attributable to limited intraosseous anastomosis of the artery in talus body, whereas inferior neck of talus is surrounded by anastomotic ring formed by the artery of canal and artery of the tarsal sinus.^[12]^ This case had a fracture line in the talus body. However, Hawkins sign appeared at 8 weeks. Tezval et al proposed that a lack of reactive posttraumatic hyperemia in the talus results in AVN^[10]^; in the present case, signs of reactive hyperemia were observed on MRI at 3 months after the surgery.

Arthrosis often occurs after talus fractures. The reported incidence of arthrosis varies from 21% to 66% and the anatomic location of the fracture corresponds to the arthritic joint.^[7]^ There was a risk of arthrosis of the tibiotalar joint and subtalar joint because our patient had fractures in the dome and posterior process of the talus body. However, no signs of arthrosis were observed as of 1 year after the operation.

There is no definitive evidence to suggest that full weight bearing contributes to the collapse of the talus. Moreover, prolonged nonweight bearing was not recommended.^[13]^ However, we did not allow weight bearing until the bone signal had changed to normal intensity on MRI because there are few studies which suggest early weight bearing for displaced talus body fracture in children.

## 4 Conclusion

The ARIF with external fixation might be the treatment of choice for such a case. Although there are no signs of AVN or arthrosis as of 1 year after the operation, we will need to follow-up the patient closely so as not to miss any complication.

## Author contributions

**Supervision:** Ryu Onishi, Masataka Yasuda, Kenta Minato, Masahiro Miyashita.

**Writing – original draft:** Shunpei Hama.

**Writing – review & editing:** Shunpei Hama.

Shunpei Hama orcid: 0000-0001-7438-5052
